# Polarization-Switchable Electrochemistry of 2D Layered Bi_2_O_2_Se Bifunctional Microreactors by Ferroelectric Modulation

**DOI:** 10.1021/acs.nanolett.4c03128

**Published:** 2024-08-26

**Authors:** Chun-Hao Chiang, Chun-Hung Yu, Yang-Sheng Lu, Yueh-Chiang Yang, Yin-Cheng Lin, Hsin-An Chen, Sheng-Zhu Ho, Yi-Chun Chen, Akichika Kumatani, Chen Chang, Pai-Chia Kuo, Jessie Shiue, Shao-Sian Li, Po-Wen Chiu, Chun-Wei Chen

**Affiliations:** † Department of Materials Science and Engineering, National Taiwan University, Taipei, 10617, Taiwan; ‡ Institute of Materials Science and Engineering, National Taipei University of Technology, Taipei, 10608 Taiwan; § Department of Electrical Engineering, National Tsing Hua University, Hsinchu, 30013, Taiwan; ∥ Department of Physics, National Cheng Kung University, Tainan, 70101, Taiwan; ⊥ Department of Electrical and Electronic Engineering, Chiba Institute of Technology, Chiba, 275-0016, Japan; # Precursory Research for Embryonic Science and Technology (PRESTO), Japan Science and Technology Agency (JST), Saitama, 332-0012, Japan; ∇ WPI-Advanced Institute for Materials Research (AIMR) and Center for Science and Innovation in Spintronics (CSIS), Tohoku University, Sendai, 980-8577, Japan; ○ Graduate School of Engineering, Tohoku University, Sendai, 980-8579, Japan; % Institute of Atomic and Molecular Science, Academia Sinica, Taipei, 10617, Taiwan; $ Center for Condensed Matter Sciences, National Taiwan University, Taipei, 10617, Taiwan; @ Center of Atomic Initiative for New Materials (AI-MAT), National Taiwan University, Taipei, 10617, Taiwan

**Keywords:** microreactors, water splitting, ferroelectric polarization, switchable electrochemistry, 2D materials

## Abstract

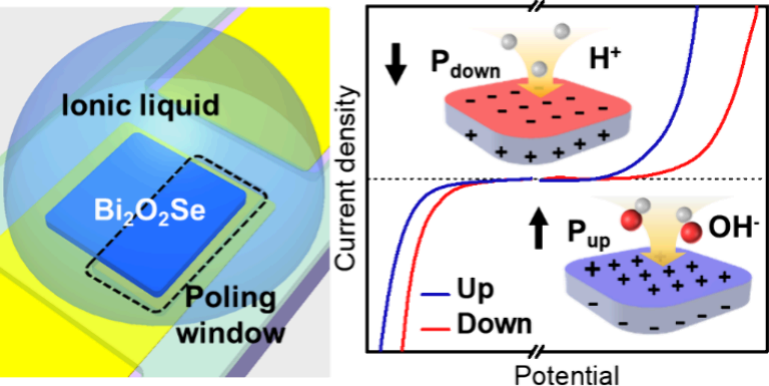

Ferroelectric catalysts are known for altering surface catalytic activities by changing the direction of their electric polarizations. This study demonstrates polarization-switchable electrochemistry using layered bismuth oxyselenide (L-Bi_2_O_2_Se) bifunctional microreactors through ferroelectric modulation. A selective-area ionic liquid gating is developed with precise control over the spatial distribution of the dipole orientation of L-Bi_2_O_2_Se. On-chip microreactors with upward polarization favor the oxygen evolution reaction, whereas those with downward polarization prefer the hydrogen evolution reaction. The microscopic origin behind polarization-switchable electrochemistry primarily stems from enhanced surface adsorption and reduced energy barriers for reactions, as examined by nanoscale scanning electrochemical cell microscopy. Integrating a pair of L-Bi_2_O_2_Se microreactors consisting of upward or downward polarizations demonstrates overall water splitting in a full-cell configuration based on a bifunctional catalyst. The ability to modulate surface polarizations on a single catalyst via ferroelectric polarization switching offers a pathway for designing catalysts for water splitting.

Electrochemical water splitting, consisting of the hydrogen evolution reaction (HER) and oxygen evolution reaction (OER), is a promising technology for sustainable energy conversion to generate clean hydrogen. Typically, a potential greater than 1.23 V is required to drive the electrochemical water splitting, where the OER process acts as the rate-limiting step and impedes the overall efficiency.^1^ The critical challenge in this technique is to pursue efficient, cost-effective, durable catalysts and enhance both cathodic HER and anodic OER with minimal overpotentials.^2^ The general strategy to design electrocatalysts with improved performances is either increasing the number of active sites or enhancing the intrinsic activity of individual active sites.^3^ In addition, external stimuli such as mechanical strain,^4^ field-effect gating,^5,6^ and magnetic field^7,8^ have shown promising effects in boosting catalytic performance. Ferroelectric catalysts, exhibiting a unique combination of an internal electric field and switchable surface chemistry, are highly desirable for tailoring surface reactivity in catalysis by manipulating the dipole orientation. For example, ferroelectric semiconductors exhibit spontaneous electric polarizations resulting from the displacement of positive and negative charges and have shown promising photocatalytic activity with an enhanced driving force for charge separation.^9^ Additionally, ferroelectric polarization offers the advantage of strong surface adsorption, which is crucial for enhancing the performance of water electrolysis.^10^

Two-dimensional (2D) layered materials such as transition-metal dichalcogenides (TMDs) have emerged as promising ultrathin catalysts for water splitting comparable to precious metals. The flat and planar characteristics of 2D layered catalysts make them exceptionally suitable for investigating the fundamental mechanisms that govern electrocatalytic reactions. Utilizing on-chip microcells/microreactors to examine 2D TMD catalysts provides a distinct advantage in directly observing spatially resolved catalytic activities of individual flakes or films.^11,12^ For example, the on-chip microreactor technique has revealed that the surface carrier concentrations of TMD semiconductor catalysts are strongly modulated by electrolyte gating during electrocatalytic reactions.^13^ Accessing these interfaces through conventional electrochemical characterizations is typically challenging, as these interfaces are generally buried. The dynamic exploration based on on-chip microreactors offers profound insights into the electronic origins of the semiconductor catalyst–electrolyte interface.^13,14^ For ferroelectric catalysts, it is known that the polarization-switching behavior significantly affects the adsorption and desorption of reactants and products on the surface of catalysts, leading to modifications in reaction pathways, kinetics, and selectivity.^15−18^ Thus, utilizing the on-chip microreactor technique to monitor polarization-dependent catalytic activities directly can provide valuable insights into surface reactivity in catalysis by manipulating the dipole orientation of ferroelectric catalysts.

In this work, we demonstrate the polarization-switchable layered bismuth oxyselenide (L-Bi_2_O_2_Se) as the bifunctional microreactor for both the HER and the OER by ferroelectric modulation. The L-Bi_2_O_2_Se has drawn significant attention recently because of its out-of-plane (OP) ferroelectricity at room temperature.^19,20^ Compared to most ferroelectric materials with large band gaps, L-Bi_2_O_2_Se shows a similar bandgap of ∼0.8 eV to silicon and high carrier mobility,^21,22^ making it unique for electronic and optoelectronic devices.^22−26^ Here, we achieve precise control over the spatial distribution of ferroelectric polarization within individual L-Bi_2_O_2_Se nanosheets for the HER and the OER using the selective-area IL gating technique. The L-Bi_2_O_2_Se nanosheets exhibit polarization-dependent electrocatalytic activities for the HER and the OER. The downward-polarized L-Bi_2_O_2_Se exhibits superior catalytic activity for the HER but inferior performance for the OER. In contrast, the upward-polarized L-Bi_2_O_2_Se exhibits catalytic activity in an opposite trend. The origins and dependence of polarization-switchable catalytic activities for HER and OER on the L-Bi_2_O_2_Se ferroelectric microreactors are unveiled using the microreactors and nanoscale scanning electrochemical cell microscopy (SECCM).^27^ Through selective-area gating, integrating L-Bi_2_O_2_Se microreactor pairs consisting of upward or downward polarizations is demonstrated for the overall water splitting in a full-cell configuration based on a bifunctional catalyst.

Figure 1a depicts the atomic structure of L-Bi_2_O_2_Se. The L-Bi_2_O_2_Se is a non-neutral layered crystal composed of charge-compensating positively charged [Bi_2_O_2_]^2+^ and negatively charged [Se]^2–^ layers along the crystallographic *c*-axis in a tetragonal conventional unit cell.^28^ In Figure 1b, the chemical vapor deposition (CVD)-grown L-Bi_2_O_2_Se nanosheets show a rectangular-shaped morphology with lateral dimensions of around tens of μm. The thickness of the L-Bi_2_O_2_Se nanosheets varies from 10 to 200 nm based on different growth parameters. It is noted that the primary objective of this work is to fabricate L-Bi_2_O_2_Se ferroelectric microreactors that are tailored for electrochemical reactions. It is essential to consider both the ferroelectric polarization switching capability and electrocatalytic characteristics of materials, as the thickness of nanosheets plays a pivotal role in influencing both aspects significantly.^13,29−31^ Thus, a thickness of ∼60 nm (Figure S1) with optimal polarization switching and electrical conductivity is chosen in this work. The Raman spectrum in Figure S2 shows an A_1g_ mode at 160.4 cm^–1^, originating from OP vibration of Bi atoms.^32^ The left panel of Figure 1c shows the scanning transmission electron microscopy-annular dark-field (STEM-ADF) image, where the tetragonal-like atomic arrangement of the crystal structure can be seen. The intensified spots arise from overlapping signals of Bi and Se atoms, confirmed by atomic-resolution energy-dispersive X-ray spectroscopy (EDS) mapping in the right panel of Figure 1c. The intensity profile indicates a Bi–Bi (or Se–Se) distance of 3.89 Å, consistent with the reported lattice spacing.^24,28,33^ The corresponding selected area electron diffraction (SAED) pattern is shown in Figure S3. Regarding the chemical states, X-ray photoelectron spectroscopy (XPS) was employed to acquire Bi 4f and Se 3d spectra, as shown in Figure S4. The Bi 4f spectrum shows two major characteristic peaks at 164.0 and 158.7 eV, assigned to 4f_5/2_ and 4f_7/2_, respectively, and associated with the chemical bonding of Bi^3+^–O_*x*_ in [Bi_2_O_2_]^2+^ layers.^26,33^ In the Se 3d spectrum, the peaks at 53.3 and 52.4 eV correspond to 3d_3/2_ and 3d_5/2_ states, respectively, from [Se^2–^] layers.^34^

**Figure 1:**
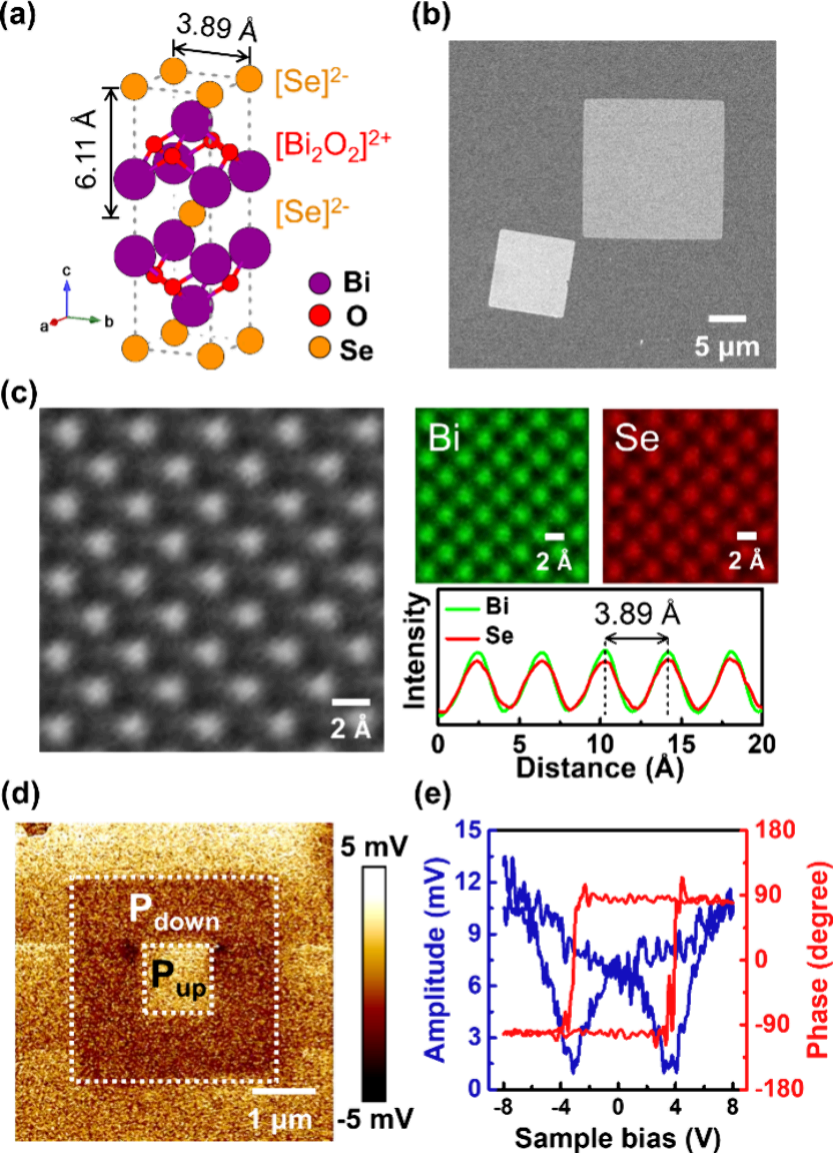
Materials characterizations on the L-Bi_2_O_2_Se nanosheets. (a) Atomic structure in a conventional tetragonal unit cell. (b) Top-view SEM image of the nanosheets on the fluorophlogopite substrate. (c) High-resolution STEM-ADF image of the L-Bi_2_O_2_Se nanosheet transferred on a lacey carbon TEM grid (left panel). Corresponding EDS mapping images and intensity line profile of Bi and Se (right panel). (d) OP PFM image with a box-in-box poling pattern in a single nanosheet. (e) PFM off-field hysteresis loop with amplitude and phase under sample bias.

The ferroelectric polarization observed in the L-Bi_2_O_2_Se nanosheets at room temperature is mainly attributed to spontaneous structural distortion breaking inversion symmetry and leading to a net OP polarization.^20,35^ To characterize the ferroelectric properties of L-Bi_2_O_2_Se, piezoresponse force microscopy (PFM) is performed to show their spontaneous polarization and switching behavior under an external electric field. The as-grown L-Bi_2_O_2_Se exhibits an upward polarization. A box-in-box pattern with downward and upward polarizations is characterized in the OP PFM image (Figure 1d) by applying a sample bias of −7 V to the outer box and +7 V to the inner box. A PFM off-field hysteresis loop displays a 180° phase switching behavior with coercive voltages around ±4 V and a butterfly like piezoresponse amplitude (Figure 1e). These results indicate the presence of spontaneous and switchable ferroelectric polarization in the L-Bi_2_O_2_Se nanosheets. In addition, the curves from contact Kelvin probe force microscopy (cKPFM) in Figure S5 exhibiting a nonlinear hysteresis loop and nonzero remnant offsets rule out the possibility of fake hysteresis from the charge injection effect.^36^

Although electric poling via a conductive tip in PFM can be achieved, it comes with limitations, such as long processing time and restricted applicability to large areas. Here, we adopt an alternative approach by using IL poling to alter the ferroelectric polarization of L-Bi_2_O_2_Se (Figure 2a). IL poling offers advantages in switching ferroelectric polarization over a large area on nanosheets. It is compatible with lithography patterning techniques to achieve selective-area modulation for polarization-switchable L-Bi_2_O_2_Se microreactors. The IL chosen for poling is diethylmethyl(2-methoxyethyl)ammonium bis(trifluoromethylsulfonyl)imide (DEME-TFSI), which is commonly employed in 2D TMD-based electronic devices to manipulate carrier concentrations, owing to its wide electrochemical stability window.^37,38^Figure 2b provides schematic diagrams of IL poling procedures. It starts from the as-grown L-Bi_2_O_2_Se nanosheet, which is transferred onto a prepatterned gold electrode. To enable selective-area poling, a poling window is created using lithography on one-half of the nanosheet, with the remaining area covered by a photoresist (PR) layer (step I). Afterward, DEME-TFSI is dropped on top of the L-Bi_2_O_2_Se. By applying a bias, the counterions drift away from the gate electrode and substantially accumulate on the surface of L-Bi_2_O_2_Se, establishing an electric field to drive polarization switching (step II). The DEME-TFSI and photoresist are removed in acetone and developer subsequently beyond completion of the gating procedure (step III). The red rectangular region marked within the L-Bi_2_O_2_Se nanosheet is further analyzed by using PFM and Kelvin probe force microscopy (KPFM). After the poling process, the corresponding OP PFM phase and work function mapping images are visualized in Figure 2c and d. The OP PFM phase contrast indicates the switching in the polarization direction through the IL poling method. The work function mapping image reveals a decrease in the work function of approximately 140 mV in the poled area with a downward polarization compared to an upward polarization. These results suggest the formation of a lateral homojunction within the L-Bi_2_O_2_Se nanosheet, with one-half displaying upward polarization and the other exhibiting downward polarization. This observation signifies the successful realization of selective-area IL poling on the L-Bi_2_O_2_Se nanosheet, which can be employed to fabricate the polarization-switchable microreactors in the following section. The origin of the electrically dependent ferroelectricity of L-Bi_2_O_2_Se is explored by density functional theory (DFT) calculations. The spontaneous polarization observed in L-Bi_2_O_2_Se is attributed to structural distortion, characterized by a relatively low energy difference between the distorted and undistorted structures.^35^ We conducted DFT calculations on the distorted Bi_2_O_2_Se lattice with symmetry breaking. Figure 2e and f depict the quantities of charge transfer along the *c*-axis direction under an electric field of ±0.05 V Å^–1^. A reversed charge redistribution under positive/negative electric fields demonstrates the switchable characteristics of the electric polarizations of L-Bi_2_O_2_Se.

**Figure 2:**
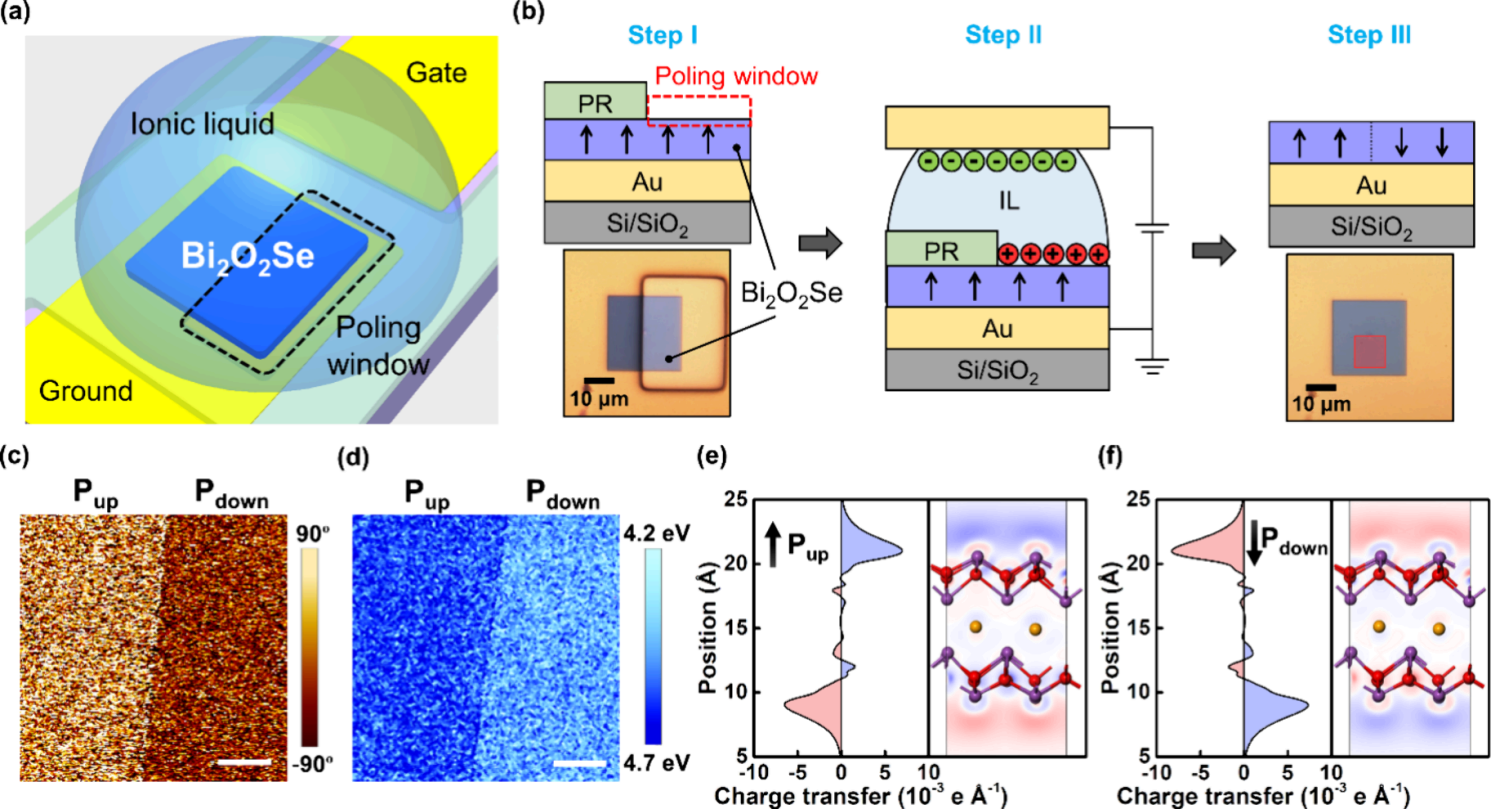
Selective-area ferroelectric modulation through IL poling. (a) Schematic diagram of IL poling on the L-Bi_2_O_2_Se nanosheet. (b) Schematic illustration for the IL poling process. The marked red area in Step III is responsible for PFM and KPFM analysis. (c) OP PFM phase and (d) work function mapping images across the pristine and poled areas within a single nanosheet after the poling process at 2.5 V for 1 h. The left is pristine, and the right is poled by the IL. Scale bar, 2 μm. (e, f) Line profiles along the *c*-axis and contour plots projected on the (100) plane of the charge redistribution under the electric field of +0.05 and −0.05 V Å^–1^, respectively. The red and blue colors represent the electron-accumulation and electron-depletion regions.

Next, on-chip ferroelectric polarization-dependent L-Bi_2_O_2_Se microreactors were fabricated to perform HER and OER by utilizing selective-area poling and microreactor fabrication techniques. The step-by-step process is schematically illustrated in Figure S6. In brief, the microreactors were designed by selecting upward- and downward-polarized regions within the precisely defined reaction window, achieved through microlithography. The exposed area in the reaction window is subjected to testing for HER and OER in acidic and alkaline conditions, respectively. The onset potentials are determined as the current density reaches 1 mA cm^–2^. The polarization curves of the HER and the OER measured by linear sweep voltammetry (LSV) are depicted in Figure 3a and b, respectively. It is found that the L-Bi_2_O_2_Se microreactor with downward polarization exhibits an enhanced HER performance, featuring a lower onset potential of −0.152 V versus reversible hydrogen electrode (RHE) compared to the −0.181 V versus RHE for the upward-polarized counterpart. In contrast, the L-Bi_2_O_2_Se microreactor with an upward polarization exhibits enhanced OER performance with the onset potentials of 1.627 V versus RHE compared to its downward-polarized counterpart with 1.710 V versus RHE. The corresponding Tafel slopes in the Figure 3a and b insets show 160 and 131 mV dec^–1^ in the HER and 175 and 215 mV dec^–1^ in the OER for upward and downward polarizations. Furthermore, electrochemical impedance spectroscopy (EIS) analyses were carried out, as shown in Figure 3c and d. The semicircle-like Nyquist plots are fitted using an equivalent circuit that includes charge-transfer resistance (*R*_ctr_) and electric-double-layer constant phase elements (*Q*_edl_) connected in parallel, which follows the established model for semiconductor catalysts.^13^ The fitting of the semicircles allows for the extraction of *R*_ctr_ and *Q*_edl_ values, as listed in Table S1. The results show that the L-Bi_2_O_2_Se microreactor with downward polarization prefers HER due to the lower *R*_ctr_ and higher *Q*_edl_ values compared to its upward-polarized counterpart. Conversely, the L-Bi_2_O_2_Se microreactor with an upward polarization tends to favor the OER compared to the downward-polarized configuration. The result demonstrates the capability to modulate surface polarizations on a single catalyst through ferroelectric switching for both the HER and the OER using the on-chip L-Bi_2_O_2_Se microreactors.

**Figure 3:**
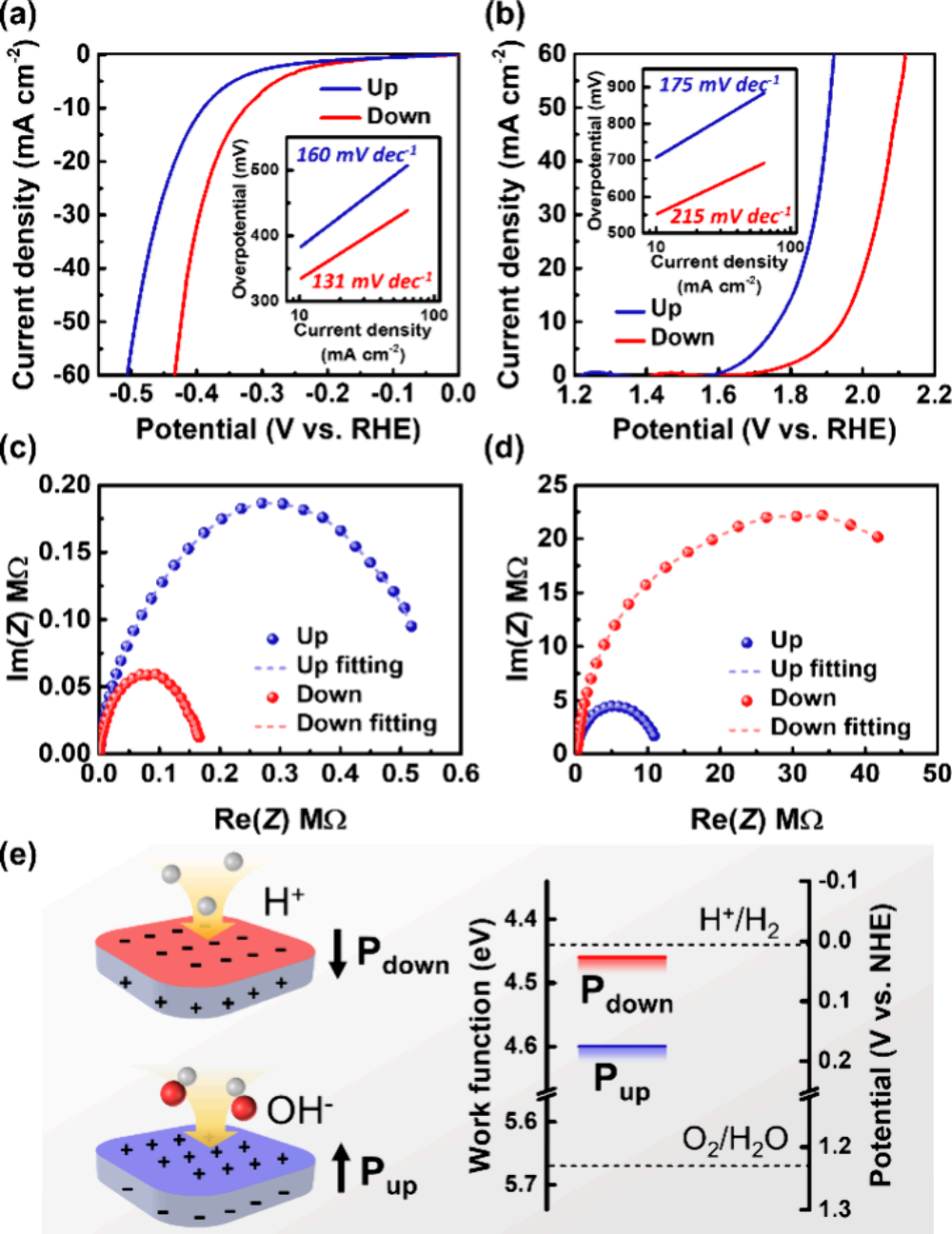
Ferroelectric polarization-dependence on the catalytic activities of the L-Bi_2_O_2_Se nanosheets in microreactor configuration. Polarization curves on the upward- and downward-polarized areas for (a) HER and (b) OER. Corresponding Tafel plots of HER and OER are in the insets. Nyquist plots from EIS analysis for (c) HER and (d) OER at the overpotentials of 400 mV. The electrolytes used are 0.5 M H_2_SO_4_ for HER and 0.1 M KOH for OER. (e) Schematic illustration for the effects of enhanced surface adsorption and reduced energy barrier on the HER and the OER.

The polarization-dependent catalytic activities of ferroelectric materials can often be explained by two key factors, band tilting and surface adsorption, which exhibit a trade-off relationship.^16^ Typically, polarization enhances surface adsorption by Coulomb attraction between the material surface and ions in the electrolyte, while the unscreened portion subsequently induces the band-tilting effect.^16^ For the L-Bi_2_O_2_Se microreactor with a downward polarization, the negatively charged surface has an advantage in attracting H^+^ cations through the Coulomb force, resulting in an enhanced *Q*_edl_ for HER. On the contrary, an upward-polarized microreactor possessing a positively charged surface gains an advantage in adsorbing OH^–^ anions or negatively charged O sites from polar H_2_O molecules.^10,16^ In addition, the L-Bi_2_O_2_Se with an upward polarization exhibits a higher work function surface than the downward counterpart, as shown in Figure 2d, which may lead to an increased energy barrier for electron transfer in HER and a relatively reduced barrier in OER (Figure 3e).^39−41^ By contrast, the L-Bi_2_O_2_Se with a downward polarization has a lower energy barrier for the HER and an increased energy barrier for the OER. Therefore, the L-Bi_2_O_2_Se microreactor with a downward polarization prefers the HER, while the L-Bi_2_O_2_Se microreactor with an upward polarization favors the OER, demonstrating the polarization-dependent surface electrochemistry of L-Bi_2_O_2_Se nanosheets. The result is similar to ultrathin 2D semiconductor catalysts, where n-type catalysts prefer cathodic reactions such as the HER, whereas p-type catalysts favor anodic reactions such as the OER.^13^ Moreover, it is worth noting that the current injection efficiency plays a vital role in the overall electrochemical performance as well, especially in the microreactor devices.^42,43^ To address this issue, conductive AFM (C-AFM) measurement was performed. The current maps of the upward- and downward-polarized L-Bi_2_O_2_Se on a gold electrode in Figure S7 show uniform values with only slight differences between the two regions. This observation highlights that the polarization-switchable surfaces dominate the overall catalytic performance of the L-Bi_2_O_2_Se microreactors.

To gain deeper insights into the microscopic origins contributing to polarization-dependent electrochemistry, we utilized SECCM to examine the spatially resolved electrochemical performance of the L-Bi_2_O_2_Se homojunction. The SECCM employs a nanopipette, a localized and mobile electrochemical cell, as a probe to offer spatial surface electrochemical characterizations in a nanoscale resolution.^27^Figure 4a shows the schematic illustration for the SECCM measurement of the L-Bi_2_O_2_Se nanosheet. A scanning nanopipette probe, which contains an electrolyte solution and a palladium (Pd) wire as a quasi-reference counter electrode (QRCE, 800 mV versus RHE^44,45^), is used in the experiment. Such a nanoprobe allows for localized discrimination and spatial visualization of electrochemical currents with a resolution of ∼80 nm. Similarly, by employing selective-area IL poling, we fabricated the L-Bi_2_O_2_Se nanosheet device with a homojunction created in opposite polarization directions. The inner square-shaped L-Bi_2_O_2_Se consists of a downward polarization, while the remaining outer L-Bi_2_O_2_Se exhibits an upward polarization. The red-marked area corresponds to the scanning area of 4 μm by 4 μm by using a nanopipet probe filled with an electrolyte of 0.5 M H_2_SO_4_ solution for the HER and 1 M phosphate buffer solution (PBS, pH 7) for the OER. To note, we used a neutral PBS solution instead of the alkaline electrolyte for the OER to prevent the glass nanopipet from etching. The LSV polarization curves for nanoscale HER and OER (Figure S8), as well as details of SECCM current mapping, are provided in the Supporting Information. Figure 4b and c presents the normalized absolute current maps measured by SECCM for HER and OER, respectively. The distribution of the local SECCM cathodic current reveals that the L-Bi_2_O_2_Se microreactor consisting of a downward polarization (square-shaped inner region) exhibits an enhanced HER performance compared to that of the upward-polarized counterpart. In addition, the downward-polarized region subjected to the IL poling process exhibits a uniform SECCM current distribution. This suggests the uniformity of polarization-switching on the L-Bi_2_O_2_Se nanosheet by the IL poling process. Conversely, the OER map exhibits enhanced local SECCM current at the outer region compared to the inner region, indicating that the upward-polarized L-Bi_2_O_2_Se favors the OER. The investigations through SECCM measurements strongly support that downward-polarized L-Bi_2_O_2_Se exhibits superior catalytic activity for HER but inferior performance for the OER. In contrast, the upward-polarized L-Bi_2_O_2_Se exhibits catalytic activity in an opposite trend. The result highlights the polarization-switchable electrochemistry of L-Bi_2_O_2_Se via ferroelectric modulation.

**Figure 4:**
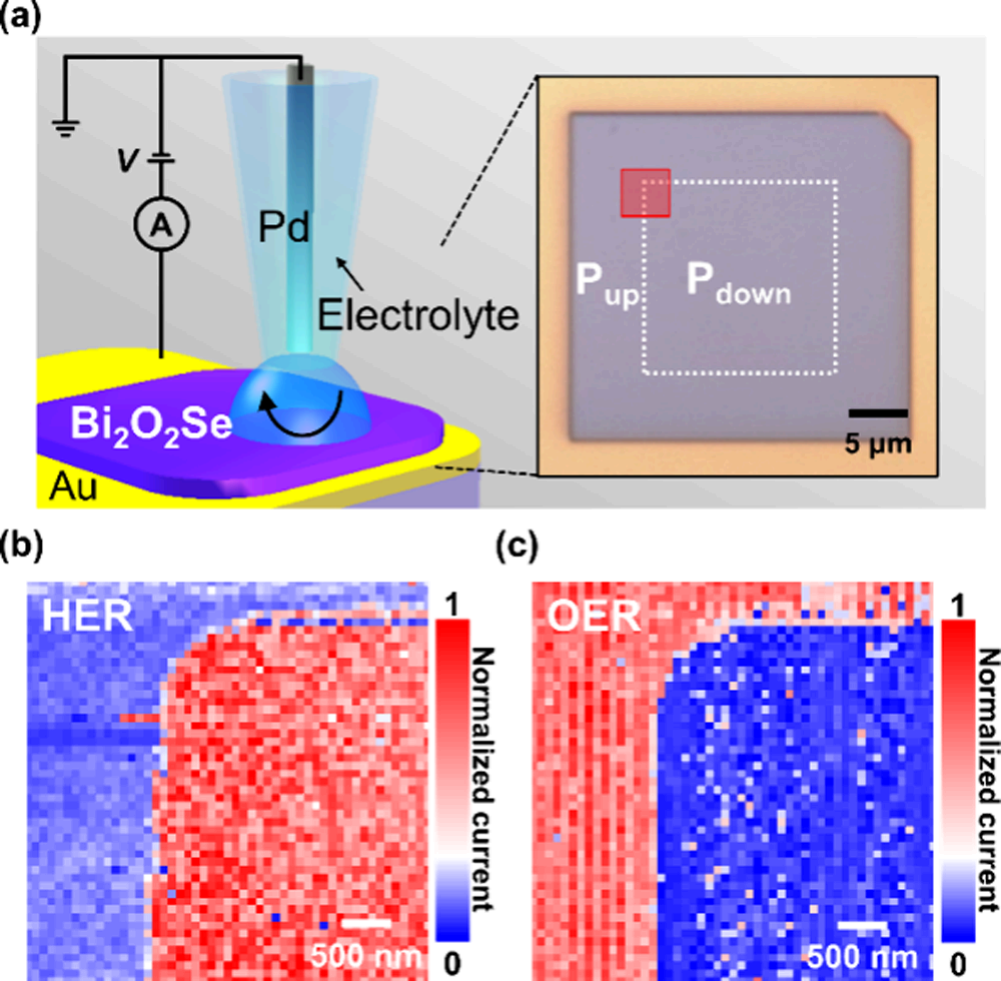
Spatially probing the catalytic activity on an IL poling-modulated L-Bi_2_O_2_Se homojunction by SECCM. (a) Schematic illustration of the SECCM measurement. The normalized absolute current maps of (b) HER and (c) OER on the marked red area in (a).

The unique switchable polarization-dependent electrochemical behaviors and bifunctional catalytic activities for the HER and the OER of L-Bi_2_O_2_Se nanosheets allow us to fabricate the paired cathode–anode microreactors for overall water splitting. As schematically depicted in Figure 5a, the L-Bi_2_O_2_Se microreactors with labels 2 and 4 are modulated to downward polarization, while the L-Bi_2_O_2_Se microreactors with labels 1 and 3 exhibit inherent upward polarization. Figure 5b displays a photograph of the paired L-Bi_2_O_2_Se microreactors when the overall water splitting was performed. By pairing the cathode and anode with combinations of “*Up–Down*”, “*Down–Down*”, “*Up–Up*”, and “*Down–Up*”, four differentiated polarization curves for overall water splitting in 1 M PBS were obtained in Figure 5c. The term “*Up–Down*” designates the cathode with upward-polarized L-Bi_2_O_2_Se and the anode with downward-polarized L-Bi_2_O_2_Se, and vice versa. The overall water-splitting performance of the paired L-Bi_2_O_2_Se microreactors shows gradual improvement with Up–Down, Down–Down, Up–Up, and Down–Up configurations. The Down–Up pair exhibits the best overall water-splitting performance with a cell voltage of 2.208 V to achieve a current density of 1 mA cm^–2^. As for the remaining pairs, extra 39, 76, and 198 mV are required for the Up–Up, Down–Down, and Up–Down pair configurations, respectively. The operation of the L-Bi_2_O_2_Se microreactors in a wide range of pH environments (pH 0 to 13) is also investigated in Figure 5d. The Down–Up pair shows enhanced performance in both acidic and alkaline conditions. In particular, it is known that the sluggish OER process, which usually acts as a bottleneck for overall water splitting, can be significantly improved in an alkaline electrolyte. Here, the Down–Up pair exhibits the best performance with a cell voltage of 2.093 V in the alkaline electrolyte, resulting from a substantial reduction in onset potential of 150 mV in OER. The bifunctional L-Bi_2_O_2_Se catalyst that can catalyze both the HER and OER simultaneously in a single electrolyte exhibits great potential in applying overall water splitting. Furthermore, the catalytic activities are known to be significantly influenced by factors such as enhanced surface reaction area, doping, or defect engineering of the materials. Therefore, it is expected that the overall water-splitting performance of bifunctional L-Bi_2_O_2_Se catalysts can be further enhanced through a synergistic combination of morphology design, doping, defect engineering, and the incorporation of ferroelectric polarizations, as proposed in this work.

**Figure 5:**
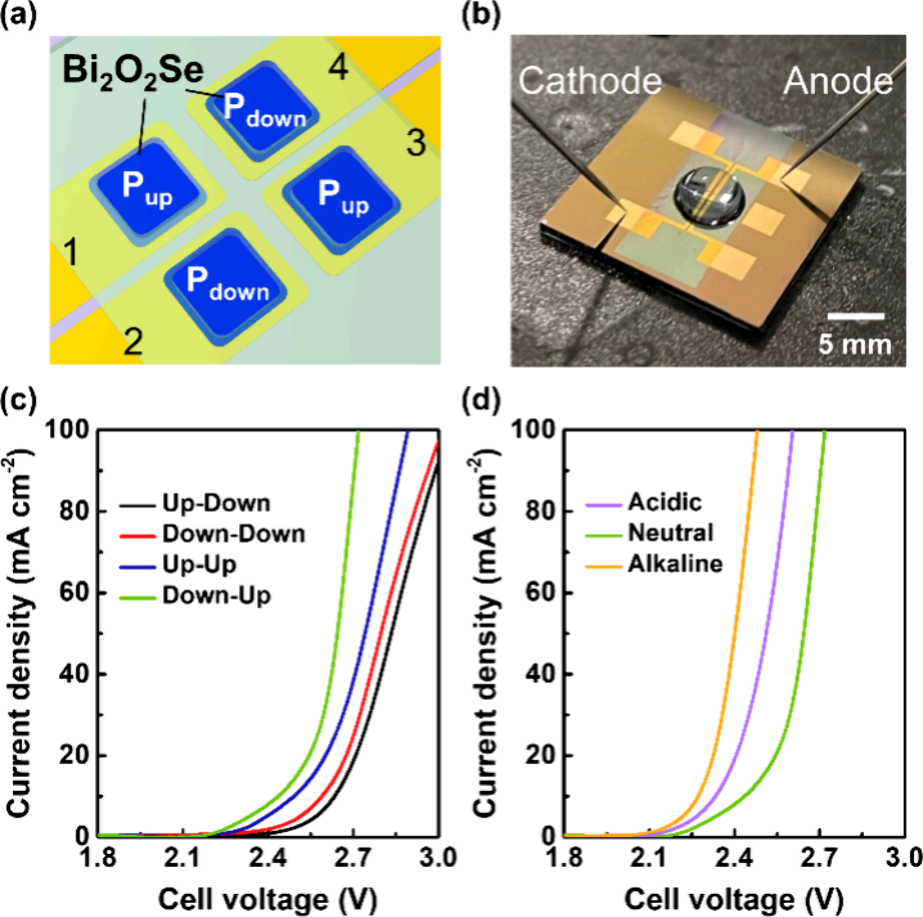
Overall water splitting microreactors based on the ferroelectric modulated L-Bi_2_O_2_Se cathode–anode pairs. (a) Schematic diagram of the overall water splitting microreactor. The L-Bi_2_O_2_Se microreactors with labels 1 and 3 are upward-polarized; the microreactors with labels 2 and 4 are downward-polarized. (b) Photograph of the microreactor while performing the water splitting. (c) Polarization curves of four ferroelectric polarization-dependent pairs in the neutral electrolyte 1 M PBS. (d) Polarization curves of the “*Down–Up*” pair to the electrolytes in a wide pH range (0.5 M H_2_SO_4_, 1 M PBS, and 0.1 M KOH).

In conclusion, this work demonstrated polarization-switchable electrochemical microreactors of L-Bi_2_O_2_Se through a controllable selective-area IL poling approach to modulate the ferroelectric polarization. A significant relationship has been investigated between ferroelectric polarizations and electrochemical behaviors associated with the HER and the OER of L-Bi_2_O_2_Se. The on-chip ferroelectric polarization-switchable microreactors of L-Bi_2_O_2_Se serve as an excellent platform for the comprehensive exploration of the fundamentals of polarization-dependent electrochemistry. These insights pave the way for future advancements in the design of efficient ferroelectric catalysts for water splitting.

## Supporting Information Available

Experimental methods; topographic image (Figure S1), Raman spectrum (Figure S2), SAED pattern (Figure S3), XPS spectra (Figure S4), and cKPFM curves (Figure S5) of L-Bi_2_O_2_Se; schematic illustration of L-Bi_2_O_2_Se microreactors (Figure S6); C-AFM current maps of L-Bi_2_O_2_Se (Figure S7); polarization curves by SECCM (Figure S8); extracted *R*_ctr_ and *Q*_edl_ values (Table S1); OP PFM images after a week (Figure S9) (PDF)
