# Supplementary material for: Polarization-Switchable Electrochemistry of 2D Layered Bi2O2Se Bifunctional Microreactors by Ferroelectric Modulation
Source: Nano Lett. 2024 Aug 26;24(35):11012–9. doi: 10.1021/acs.nanolett.4c03128 (PMC11378338; doi:10.1021/acs.nanolett.4c03128)
Supplement: Supplementary file 1 — nl4c03128_si_001.pdf [file nl4c03128_si_001.pdf]

Supporting information

Polarization-Switchable Electrochemistry of 2D Layered Bi_2O_2Se Bifunctional Microreactors by Ferroelectric Modulation

Chun-Hao Chiang,[1,‡] Chun-Hung Yu,[1,‡] Yang-Sheng Lu,[2] Yueh-Chiang Yang,[3] Yin-Cheng Lin,[1] Hsin-An Chen,[2] Sheng-Zhu Ho,[4] Yi-Chun Chen,[4] Akichika Kumatani,[5,6,7,8] Chen Chang,[1] Pai-Chia Kuo,[9] Jessie Shiue,[9] Shao-Sian Li,[2,] Po-Wen Chiu,[3,9] and Chun-Wei Chen,[1,10,11,*]*

[1]Department of Materials Science and Engineering, National Taiwan University, Taipei 10617, Taiwan

[2]Institute of Materials Science and Engineering, National Taipei University of Technology, Taipei, 10608 Taiwan

[3]Department of Electrical Engineering, National Tsing Hua University, Hsinchu 30013, Taiwan

[4]Department of Physics, National Cheng Kung University, Tainan, 70101, Taiwan

[5]Department of Electrical and Electronic Engineering, Chiba Institute of Technology, Chiba, 275-0016, Japan

[6]Precursory Research for Embryonic Science and Technology (PRESTO), Japan Science and Technology Agency (JST), Saitama, 332-0012, Japan

[7]WPI-Advanced Institute for Materials Research (AIMR) and Center for Science and Innovation in Spintronics (CSIS), Tohoku University, Sendai, 980-8577, Japan

[8]Graduate School of Engineering, Tohoku University, Sendai, 980-8579, Japan

[9]Institute of Atomic and Molecular Science, Academia Sinica, Taipei 10617, Taiwan

[10]Center for Condensed Matter Sciences, National Taiwan University, Taipei, 10617, Taiwan

[11]Center of Atomic Initiative for New Materials (AI-MAT), National Taiwan University, Taipei, 10617, Taiwan

EXPERIMENTAL METHODS

Materials characterizations. The SEM image is collected through NOVA NANO SEM 450. The Raman spectrum is acquired by an Andor Kymera 193i-B2 spectrometer and an Andor iDus416 low-noise detector under an Olympus optical microscope (50X objective lens). The excitation laser is a 532-nm Nd:YAG continuous-wave laser. The L-Bi_2O_2Se on a heavily doped Si substrate is prepared for XPS analysis. The XPS spectra are collected on a Thermo Scientific Theta Probe with an aluminum anode. The L-Bi_2O_2Se is transferred onto a lacey carbon copper grid for the TEM and STEM characterizations. The corresponding images, EDS mapping, and SAED pattern are performed on JEOL JEM-ARM300F2.

KPFM, C-AFM, PFM, cKPFM, and hysteresis loop. KPFM and C-AFM were performed by using conductive Pt/Ir-coated tips on the Innova system (Bruker). The work functions of Bi_2O_2Se were calculated based on the work function of Au (5.1 eV) and measured surface potentials. PFM results were collected by using a commercial scanning probe microscope system (Multimode 8, Bruker) with a Nanoscope Controller V. The PFM signal image, off-field hysteresis loops, and cKPFM curves were all carried out under contact-resonance mode with the commercial Pt/Ir-coated tips with a spring constant of 2.8 N m^{-1} (NANOSENSORS PPP-EFM60). The tip was driven with an AC voltage amplitude of about 1 V and was working at a contact-resonance frequency of about 300 kHz. The 5 μm × 5 μm box-in-box PFM signal image was captured after first poled by -7 V sample bias in a 3 μm × 3 μm area and then poled by 7 V in a 1 μm × 1 μm area to flip the polarization from downward to upward direction. The off-field hysteresis data and cKPFM curves

were obtained via the switching spectroscopic technique with an arbitrary waveform generator (G5100A, Picotest).

CVD growth of the Bi_2O_2Se nanosheets. To begin, precursors Bi_2O_3 (10 mg, 99%, Alfa Aesar) and Bi_2Se_3 (10 mg, 99.995%, Merck) were arranged in a checkerboard pattern on a quartz boat carrier, with fluorophlogopite (1 x 1cm^2) positioned 0.1 cm directly above the precursors. Subsequently, the quartz boat was inserted into a 1-inch quartz tube at the center of the furnace for the synthesis of L-Bi_2O_2Se using the vertical reaction method. During the growth stage, the temperature was initially set to 100˚C, and 300 sccm of argon gas was introduced for 30 minutes to eliminate water and purge any remaining gases. The heating rate was then maintained at 50˚C min^{-1}, while the pressure was held at 100 torr. Upon reaching the growth temperature of 570˚C, the argon flow was adjusted to 100 sccm, and the growth proceeded for 15 minutes. Following the completion of the growth process, the tube was promptly removed from the furnace. The temperature was rapidly reduced at a rate exceeding 100˚C min^{-1}, while the argon flow was adjusted to 300 sccm. Finally, the samples were retrieved and immediately placed in a drying box to prevent moisture adsorption.

PS-assisted transfer method. The polystyrene (PS) solution is prepared in 9 wt% (molecular weight of 2.5 x 10^5) dissolved in toluene. The PS solution is spin-coated on the as-grown L-Bi_2O_2Se sample (600 rpm for 10 s and 2000 rpm for 60 s) and dried on a hotplate at 70 ˚C for 15 min as a supporting and sacrificial layer. Detachment of the L-Bi_2O_2Se relies on the water penetration at the interface of PS film and mica from scratch created intentionally at the sample edge. The PS film then gradually peeled off and floated on the water surface. Target substrates, including TEM

grid, Si, and $Si/SiO_2/Au$, are used to fish up the floating L-Bi_2O_2Se/PS, followed by baking at 150 °C to remove residual water. Lastly, the PS film is dissolved in toluene for at least three batches, leaving the transferred L-Bi_2O_2Se on the target substrate.

Ionic liquid poling. The tiny amount (~10 μl) of the IL is dropped onto the sample patterned by micro-lithography. Before applying the bias, the ionic liquid and chip are placed in a vacuum chamber (Lake Shore) overnight to minimize water adsorption in the ionic liquid. The entire poling process is carried out under vacuum conditions (~3 x 10^{-3} torr). The voltage is applied and controlled by using a Keithley 2400 sourcemeter and Labview program.

DFT calculations. In this research, ab initio calculations of L-Bi_2O_2Se were performed using the Vienna Ab initio Simulation Package (VASP) based on density functional theory (DFT).[1-3] The generalized gradient approximation (GGA) was used with the Perdew-Burke-Ernzerhof (PBE)[4, 5] exchange-correlation functional and the projector augmented wave (PAW)[6, 7] pseudopotentials. The cutoff energy was set to be 400 eV, and the self-consistent field convergence criterion for energy was set to be 10^{-6} eV. The electronic structure was fully optimized until the total energy change between steps was smaller than 10^{-5} eV. To present the spontaneous fluctuations of atoms under room temperature, an ab-initio molecular dynamics simulation was performed using NVT ensemble regulated by a Nose-Hoover thermostat. The temperature was maintained at 300 K. 4 x 4 x 1 Γ-centered k-point mesh was used. The simulation time step was set to be 0.5 fs. To model the van der Waals interaction between layers, DFT-D3 was incorporated into all calculations.[8]

Fabrication of the microreactors. The metal electrodes (5 nm Cr/50 nm Au) on Si/SiO_2 substrates for L-Bi_2O_2Se microreactors are defined using a metal shadow mask and deposited by a thermal evaporator. The L-Bi_2O_2Se is transferred on top of the metal electrodes through the PS-assisted transfer method. The reaction windows to expose the electrolytes are defined via micro-lithography with a layer of photoresist, which acts as a strong electrochemically blocking layer.

Electrochemical measurements of the microreactors. The electrochemical currents and EIS analysis are performed using Metrohm Autolab PGSTAT204 with the FRA32M EIS module. The measurements are performed in a Faraday cage to prevent the influence of external electromagnetic interference. For HER and OER, the reference electrode is the Ag/AgCl electrode, and the counter electrode is the platinum wire. A tungsten probe controlled by XYZ micropositioner is used to contact the metal electrodes of L-Bi_2O_2Se nanosheets, which act as working electrodes. For overall water splitting, the two tungsten probes are used to contact two L-Bi_2O_2Se electrodes. The electrolytes are purged by Ar gas for 30 min before measurements. All the polarization curves are performed without iR correction. The EIS analysis is measured in a frequency ranging from 10^6 to 0.01 Hz and an amplitude of 5 mV.

SECCM measurements. We employed SECCM to assess the catalytic performance of L-Bi_2O_2Se nanosheets in water splitting. A Pd wire as QRCE with a diameter of approximately 250 nm was inserted into silicate capillaries about 70 nm in diameter, filled with electrolytes, serving as a quasi-reference counter electrode. The potential of the L-Bi_2O_2Se (as a working electrode) was controlled relative to the Pd QRCE at the tip, while the current was monitored with a variable-gain transimpedance amplifier. In this work, the LSV scan rate was set at 100 mV s^{-1}, and the

measurement time was 20 ms per point. The imaging time was typically 40 min for the imaging resolution of 128 x 128 pixels in a 10 μm x 10 μm area. The current mapping of HER and OER were obtained at potentials of -1.7 V and 1.4 V versus Pd (equivalent to the potentials of -0.9 V and 2.2 V versus RHE), respectively.

SUPPLEMENTARY FIGURES

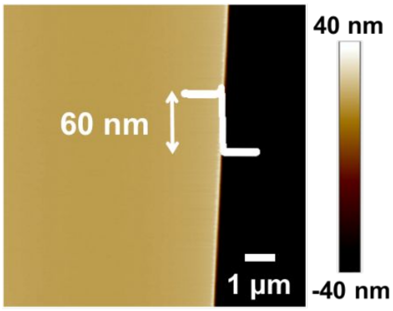

Figure S1. Topographic image of the L-Bi_2O_2Se nanosheet with a thickness of 60 nm.

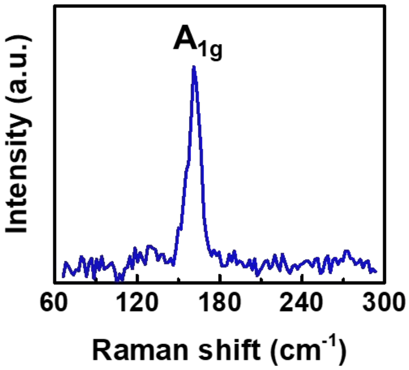

Figure S2. Raman spectrum of the as-grown L-Bi_2O_2Se nanosheet..

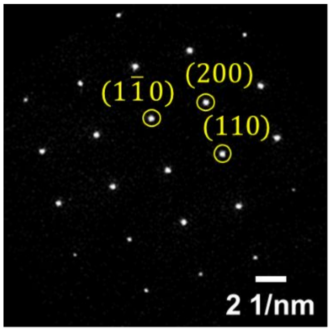

Figure S3. SAED pattern from the zone axis [001] of the L-Bi_2O_2Se nanosheet, which is transferred on a lacey carbon TEM grid.

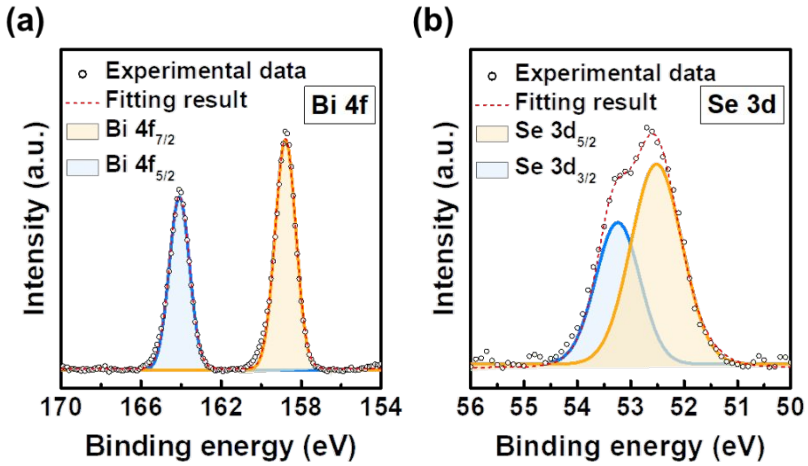

Figure S4. XPS (a) Bi 4f and (b) Se 3d spectra of the as-grown L-Bi_2O_2Se nanosheets.

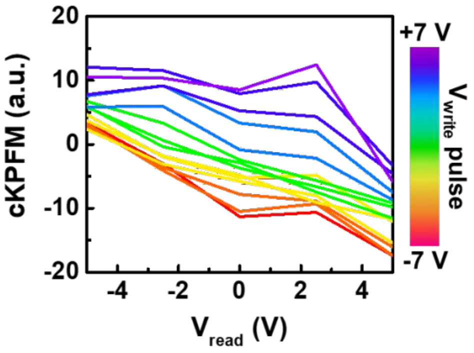

Figure S5. cKPFM curves performed on the L-Bi_2O_2Se nanosheet as a function of V_{read} under a series of V_{write}.

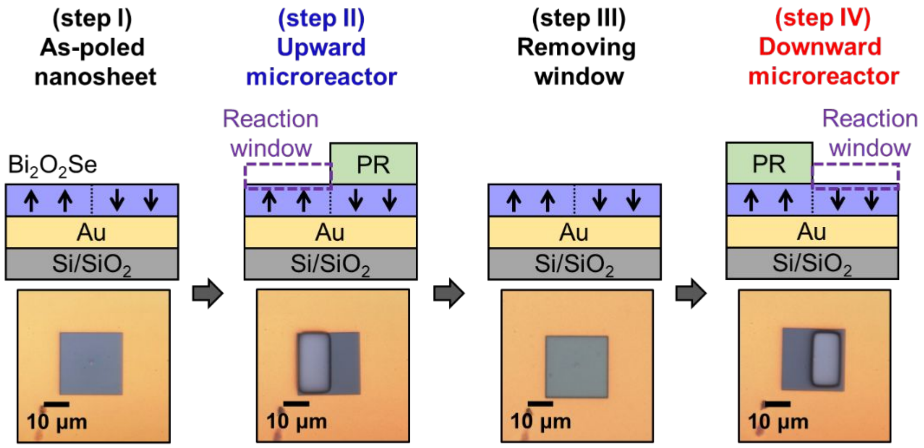

Figure S6. Schematic illustration for the L-Bi_2O_2Se microreactors fabrication on the upward and downward-polarized areas.

		R_{ctr} (MΩ)	Q_{edl} (nS·s^N)
HER	Up	0.58	19.5 (N = 0.995)
	Down	0.17	26.4 (N = 0.996)
OER	Up	11.4	8.14 (N = 0.998)
	Down	58.5	3.12 (N = 0.997)

Table S1. The extracted R_{ctr} and Q_{edl} values from the fitting of an equivalent circuit. The equivalent circuit consists of the R_{ctr} and Q_{edl} in parallel.

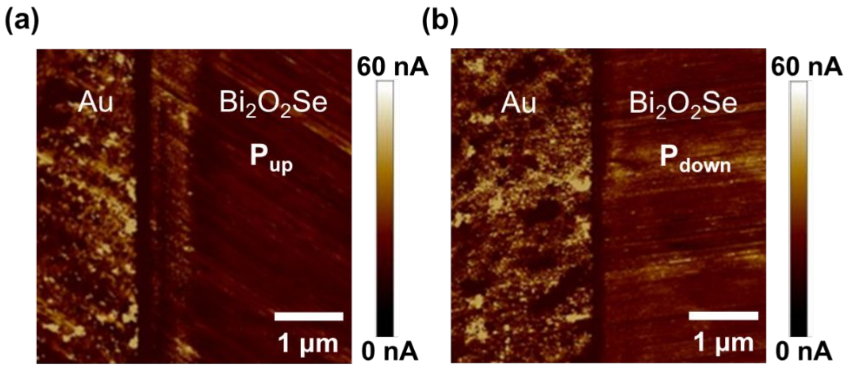

Figure S7. The current maps of (a) upward and (b) downward-polarized L-Bi_2O_2Se on gold electrode measured by C-AFM.

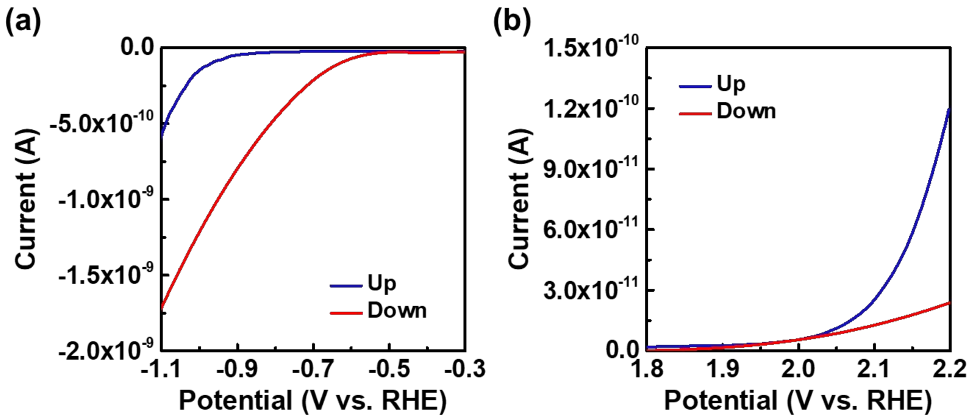

Figure S8. Polarization curves measured by the pinned nanopipette of the SECCM for (a) HER in 0.5 M H_2SO_4 and (b) OER in 1 M PBS.

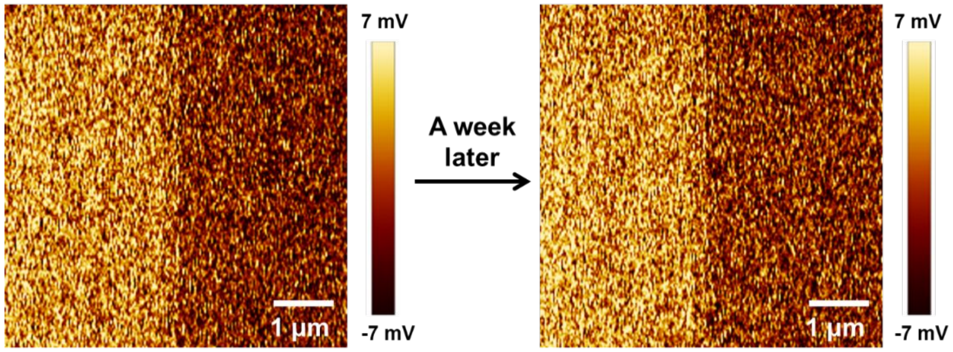

Figure S9. The OP PFM image with upward and downward areas after a week. The sample is kept in a dry box after poling. There is no obvious change observed between as-poled and a-week-later images.

REFERENCES

(1) Kresse, G.; Hafner, J. Ab initio molecular dynamics for liquid metals. *Phys. Rev. B* **1993**, *47*, 558-561.

(2) Kresse, G.; Hafner, J. Ab initio molecular dynamics for open-shell transition metals. *Phys. Rev. B* **1993**, *48*, 13115-13118.

(3) Kresse, G.; Hafner, J. Ab initio molecular-dynamics simulation of the liquid-metal-amorphous-semiconductor transition in germanium. *Phys. Rev. B* **1994**, *49*, 14251-14269.

(4) Perdew, J. P.; Chevary, J. A.; Vosko, S. H.; Jackson, K. A.; Pederson, M. R.; Singh, D. J.; Fiolhais, C. Atoms, molecules, solids, and surfaces: Applications of the generalized gradient approximation for exchange and correlation. *Phys. Rev. B* **1992**, *46*, 6671-6687.

(5) Perdew, J. P.; Wang, Y. Accurate and simple analytic representation of the electron-gas correlation energy. *Phys. Rev. B* **1992**, *45*, 13244-13249.

(6) Blochl, P. E. Projector augmented-wave method. *Phys. Rev. B* **1994**, *50*, 17953-17979.

(7) Kresse, G.; Joubert, D. From ultrasoft pseudopotentials to the projector augmented-wave method. *Phys. Rev. B* **1999**, *59*, 1758-1775.

(8) Grimme, S.; Antony, J.; Ehrlich, S.; Krieg, H. A consistent and accurate ab initio parametrization of density functional dispersion correction (DFT-D) for the 94 elements H-Pu. *J. Chem. Phys.* **2010**, *132*, 154104.
